# *In vitro* efficacy of humanized regimen of flomoxef against extended-spectrum β-lactamase-producing *Escherichia coli* and *Klebsiella pneumoniae*

**DOI:** 10.1128/aac.00258-23

**Published:** 2023-08-23

**Authors:** Hidenori Yamashiro, Yu Kasamatsu, Naomi Anan, Miki Takemura, Yoshinori Yamano

**Affiliations:** 1 Laboratory for Drug Discovery and Disease Research, Shionogi & Co., Ltd., Toyonaka, Osaka, Japan; 2 Department of Infection Control and Laboratory Medicine, Kyoto Prefectural University of Medicine, Kyoto, Japan; University of Pittsburgh, Pittsburgh, Pennsylvania, USA

**Keywords:** extended-spectrum beta-lactamases, antimicrobial, *in vitro* chemostat model, flomoxef, carbapenem sparing

## Abstract

This study compared the efficacy of flomoxef with other β-lactam antibiotics against extended-spectrum β-lactamases (ESBL)-producing bacteria of clinical relevance. First, the prevalence and β-lactamase genotypes of ESBL-producing strains among *Escherichia coli* and *Klebsiella pneumoniae* isolates collected in Japan from 2004 to 2018 were investigated. High MIC_90_ values (>64 µg/mL) of ceftriaxone, cefepime, and ceftazidime and low MIC_90_ values (≤0.06–2 µg/mL) of flomoxef, cefmetazole, and meropenem against both species were observed. Second, a chemostat model was used to analyze the efficacy of humanized regimens of three oxacephem/cephamycin antibiotics (flomoxef, cefmetazole, cefoxitin) and two other antibiotics (meropenem and piperacillin/tazobactam) in suppressing the growth of five ESBL-producing *E. coli* and two *K. pneumoniae* strains. Flomoxef, piperacillin/tazobactam, and meropenem showed good bactericidal effects with >4 log_10_ CFU/mL reduction without bacterial regrowth at 24 h even when the MIC of test isolates was >MIC_90_. Cefmetazole and cefoxitin resulted in regrowth of test isolates with MIC ≥MIC_90_ at 24 h. Cefmetazole, cefoxitin, flomoxef, and meropenem showed increased MICs for regrown samples. A clear relationship between the proportion of time that the free drug concentration exceeded the MIC (%*f*T_>MIC_) and antibiotic efficacy was found for flomoxef, cefoxitin, and cefmetazole, and flomoxef had the highest %*f*T_>MIC_, whereas discrepancies between Clinical and Laboratory Standards Institute breakpoint and bactericidal activity were observed for cefmetazole. Flomoxef was effective in preventing the growth of all ESBL-producing strains, even those with an MIC eight times the MIC_90_. Thus, flomoxef may be a good alternative to meropenem in context of carbapenems sparing stewardship.

## INTRODUCTION

The emergence and spread of extended-spectrum β-lactamases (ESBL) among human pathogens have become a serious public health concern worldwide, narrowing the therapeutic options for the treatment of hospital and community-acquired infections (1). ESBL hydrolyze some commonly used β-lactam antibiotics, including penicillin and cephalosporins, and make these drugs ineffective for treating infections. ESBL-producing Enterobacterales have become major multidrug-resistant pathogens in the last two decades, and the multifactorial nature of its expansion poses a major challenge in the efforts to control them (1). Mobile genetic elements are thought to be responsible for ESBL spread (2), and ESBL-producing bacteria are associated with increased mortality rates, longer hospital stays, and increased costs for healthcare systems (3, 4). Therefore, finding adequate therapies to eradicate these infectious agents has become critical.

Previously, TEM- and SHV-type ESBL were the predominant families of ESBL. Currently, cefotaxime (CTX)-M type enzymes are the most commonly found ESBL type with the CTX-M-15 variant being the most prevalent worldwide (5). The increasing prevalence of CTX-M-type ESBL-producing bacteria has become a serious problem. Additionally, acquired AmpC-type β-lactamases are clinically important cephalosporinases for some species of Enterobacterales, as they mediate resistance to cephalothin, cefazolin, cefoxitin, penicillin, and β-lactam/β-lactamase inhibitor (such as amoxicillin/clavulanic acid, ampicillin/sulbactam, and piperacillin/tazobactam) combinations. The treatment options for infections caused by ESBL-producing pathogens are limited because they are resistant to many commonly prescribed antibiotics. Therefore, the antimicrobials effective against ESBL-producing microorganisms need to be developed and tested. The urgency of the development of these antimicrobials was indicated in a pathogens list previously published by the World Health Organization, wherein they have been included in the “critical” category (https://www.who.int/publications/i/item/WHO-EMP-IAU-2017.12) and in an antibiotic resistance threats list published by the Centers for Disease Control and Prevention as one of the serious threats (https://www.cdc.gov/drugresistance/biggest-threats.html).

Typically, the first-line choice against ESBL-producing bacteria is a carbapenem (6). However, carbapenem-sparing approaches are gaining acceptance in the context of antimicrobial stewardship programs, as the overuse of these agents has been linked to the development of resistance against carbapenems (7). It has been reported that cephamycins and oxacephems are not degraded by ESBLs (8); therefore, they may be valid options for treating infections caused by ESBL-producing microbes.

The present study aimed to compare, using an *in vitro* chemostat model, the efficacy of flomoxef (FMOX), an oxacephem antibiotic, and cephamycins against ESBL-producing bacteria of clinical relevance to assess appropriate treatment options other than carbapenems. FMOX was first synthesized in Japan in 1980s and is currently marketed only in East Asia such as Japan, China, South Korea, and Taiwan (9, 10). FMOX is widely active against Gram-positive, Gram-negative, and anaerobic bacteria, and its activity against ESBL-producing bacteria has recently attracted attention (8, 10 - 13). The clinical isolates used in this research were collected during a multicenter study conducted in Japan from 2004 to 2018.

## RESULTS

### Prevalence of ESBL-producing bacteria and major betaβ-lactamase families

**Fig 1:**
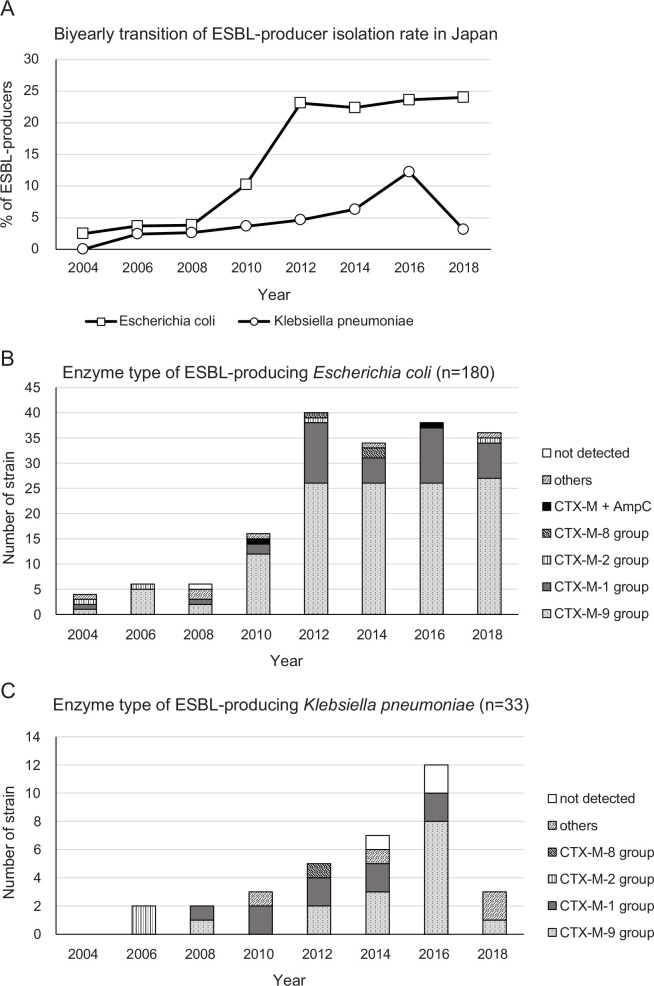
(A) Prevalence of extended-spectrum β-lactamases (ESBL)-producing *Escherichia coli* and *Klebsiella pneumoniae* in Japan. (B) Types of enzymes in ESBL-producing *E. coli*. (C) Types of enzymes in ESBL-producing *K. pneumoniae*.

Among *Escherichia coli* clinical isolates, the prevalence of ESBL-producing strains was revealing a strong increase from 2008 to 2012 (Fig. 1A). The rate of *Klebsiella pneumoniae* isolates producing ESBL also increased during the period analyzed, but much more slowly, and decreased again in 2018 to reach a value similar to that before 2010. Considering the whole 14-y period analyzed, the frequency of ESBL-producing strains was 14.1% (180/1274) for *E. coli* and 4.6% (33/720) for *K. pneumoniae*.

Both ESBL-producing isolates of *E. coli* and *K. pneumoniae* mostly belonged to the CTX-M-9 group, followed by the CTX-M-1 group (Fig. 1B and C).

Table 1 shows the susceptibility profile of these ESBL-producing isolates for several antibiotics, and includes the minimum inhibitory concentration (MIC)_50_ and MIC_90_ values calculated for each drug. While the high MIC_90_ values (>64 µg/mL) of ceftriaxone (CTRX), cefepime (CFPM), and ceftazidime (CAZ) against both species were observed, FMOX, cefmetazole (CMZ), cefoxitin (CFX), and meropenem (MEPM) showed lower MIC_90_ values against both species (0.25–0.5 µg/mL, 4 µg/mL, 16–32 µg/mL, and ≤0.06 µg/mL, respectively). Of note, among 180 ESBL-producing *E. coli* isolates, only two were AmpC co-producers, and no *K. pneumoniae* isolate displayed this feature. Though rare, these strains were resistant to cephems and cephamycins, showing relatively high MICs for these antimicrobials. One of these strains simultaneously produced CTX-M-1 and DHA, and the other one produced CTX-M-9 and CMY; both have been included as “CTX-M +AmpC” in Fig. 1B. “Others” in Fig. 1B include other ESBL (SHV) producers, ESBL co-producers (CTX-M-1 group and CTX-M-9 group, CTX-M-9 group and TEM-ESBL, and CTX-M-1 group and SHV-ESBL), and co-producers of CTX-M-1 group and OXA-1. Regarding FMOX, two isolates with high MIC, namely, CMY-type AmpC-producing *E. coli* (MIC >64 µg/mL) and DHA-type AmpC-producing *E. coli* (MIC 8 µg/mL), were AmpC producers.

**TABLE 1:** MIC distribution against extended-spectrum β-lactamases-producing *Escherichia coli* and *Klebsiella pneumoniae* isolated in Japan between 2004 and 2018^*b*^

	MIC (μg/mL)												MIC_50_	MIC_90_		
Antibiotics	≤0.06	0.12	0.25	0.5	1	2	4	8	16	32	64	>64	(μg/ mL)	(μg/ mL)	%S^*a*^	%R^*a*^
ESBL-producing *E. coli* isolated between 2004 and 2018 (180)^*b*^																
Ceftazidime	0	0	0	4	15	26	27	45	19	13	10	**21**	8	>64	40.0	35.0
Ceftriaxone	0	0	0	0	0	0	0	0	1	0	4	**175**	>64	>64	0	100
Cefepime	0	0	1	1	1	4	15	34	26	24	11	**63**	32	>64	3.9	68.9
Flomoxef	49	76	30	**14**	5	2	0	2	0	1	0	1	0.12	0.5	NA	NA
Cefmetazole	0	0	0	8	72	53	**29**	13	2	1	2	0	2	4	98.3	1.1
Cefoxitin	0	0	0	0	1	12	73	55	**24**	8	5	2	8	16	78.3	8.3
Piperacillin/tazobactam	0	0	0	0	8	86	47	12	**14**	3	6	4	2/4	16/4	85.0	7.2
Meropenem	**179**	1	0	0	0	0	0	0	0	0	0	0	≤0.06	≤0.06	100	0
ESBL-producing *K. pneumoniae* isolated between 2004 and 2018 (33)^*b*^																
Ceftazidime	1	0	0	4	5	5	3	2	3	3	3	**4**	4	>64	54.5	39.4
Ceftriaxone	1	0	0	0	1	0	0	2	0	1	4	**24**	>64	>64	3.0	93.9
Cefepime	1	0	0	2	3	4	3	5	3	0	3	**9**	8	>64	30.3	45.5
Flomoxef	16	10	**5**	1	0	0	1	0	0	0	0	0	0.12	0.25	NA	NA
Cefmetazole	0	0	0	7	15	4	**5**	1	0	1	0	0	1	4	97.0	0
Cefoxitin	0	0	0	0	0	14	7	4	4	**3**	1	0	4	32	75.8	12.1
Piperacillin/tazobactam	0	0	0	0	2	9	6	5	6	**2**	0	3	4/4	32/4	66.7	15.2
Meropenem	**33**	0	0	0	0	0	0	0	0	0	0		≤0.06	≤0.06	100	0

^*a*^ %S and %R were defined based on CLSI guideline M100-Ed32 (15).

^*b*^ ESBL, extended-spectrum beta-lactamases; CLSI, Clinical and Laboratory Standards Institute; S, susceptible; R, resistance; NA, not available; underlined numbers, MIC_50_; bold numbers, MIC_90_.

### Antimicrobial effects on selected ESBL-producing isolates assessed by a chemostat assay

We used five *E. coli* and two *K. pneumoniae* isolates that produced CTX-M-type ESBL to carry out the chemostat assays; Table S2 shows the main characteristics of these seven strains. FMOX and two cephamycin antibiotics, CMZ and CFX, were evaluated because oxacephems and cephamycins are considered to be possible option as carbapenem-sparing therapy due to their activity against ESBL producers (6, 15). MEPM and piperacillin/tazobactam (PIPC/TAZ) were used as a control to compare the potential as carbapenem-sparing therapy. It may be observed that isolates had MIC values similar to those representing MIC_90_ values for each antibiotic during the entire period of analyses (2004–2018). However, to observe the efficacy of FMOX with MIC above MIC_90_, we also included one strain of *E. coli,* SR43056, with MIC value of 4 µg/mL, which is eight-fold higher than the MIC_90_ for FMOX. Additionally, a strain of *K. pneumoniae,* SR34688, was used as a positive control to confirm the efficacy of each antibiotic against susceptible isolates.

The daily time course of antibiotic concentrations (free form) in human plasma when administered at their standard regimes and the pharmacokinetic parameters used to predict them is shown in Fig. S1. All drugs peaked at about 1 h after each dose due to the 1 h infusion regimen, and then the concentration diminished slowly up to the next dose. The difference in the free concentration of antibiotics may be partly due to the differences in their protein-binding properties. FMOX, CMZ, and CFX showed similar concentration–time curves; however, FMOX reached higher free concentration levels (μg/mL) than did the other cephamycins.

**Fig 2:**
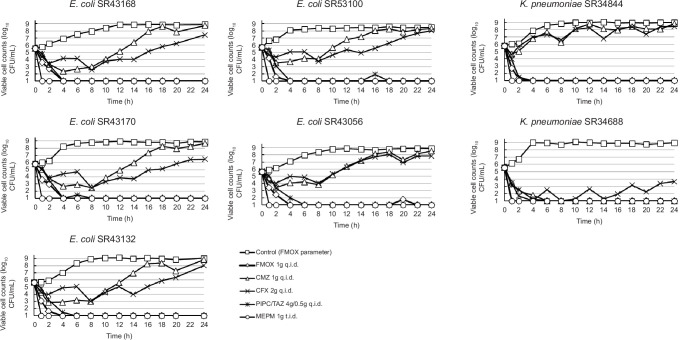
Time course of viable cell counts for seven extended-spectrum β-lactamase-producing clinical isolates in the presence of different antibiotics

Fig. 2 summarizes the time–kill curves under humanized pharmacokinetic regimens in the chemostat assay. Under human pharmacokinetic reproducible conditions, FMOX 1 g q.i.d. showed good bactericidal effects with >4 log_10_ CFU/mL reduction and no regrowth at 24 h for all isolates with MIC ≤4 µg/mL. For CFX 2 g q.i.d. and CMZ 1 g q.i.d., bactericidal activity at 24 h was achieved only for one isolate with lower MIC that was used as a positive control and was not achieved for all other test isolates with MIC close to MIC_90_ (16–32 µg/mL for CFX and 4–16 µg/mL for CMZ). It should be noted that these isolates are defined as intermediate or resistant to CFX, but susceptible to CMZ based on Clinical and Laboratory Standards Institute (CLSI) interpretation based on CLSI guideline M100-Ed32 (14).

**Fig 3:**
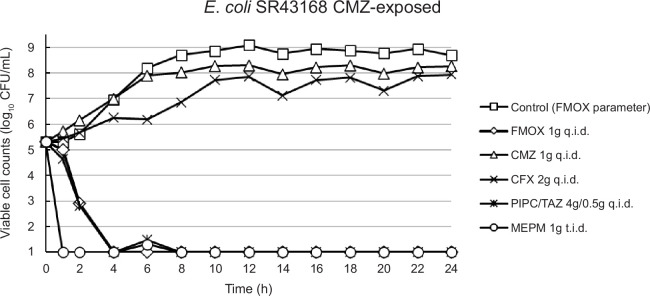
Time course of viable cell counts for cefmetazole-exposed strain in the presence of different antibiotics. FMOX, flomoxef; CMZ, cefmetazole; CFX, cefoxitin; PIPC/TAZ, piperacillin/tazobactam; MEPM, meropenem.

From the regrown samples, some colonies were picked up and the MICs of several antibiotic classes were determined through standard broth microdilution method (Table S3). Both CMZ and CFX-exposed *E. coli* strains showed elevations not only in the MIC values of CMZ and CFX, but also for other β-lactam drugs to which the parent strains had not been exposed, including FMOX and MEPM. Although MIC values of FMOX and MEPM for the CMZ-exposed strain were as high as 4 µg/mL and 0.5 µg/mL, respectively, the maximum clinical dose of FMOX recreated in this model was shown to have a strong antibacterial effect against this strain (Fig. 3).

### Bactericidal effect and %*f*T_>MIC_

The overall results from our chemostat assay are summarized in Table 2. A clear relationship between the proportion of time that the free (unbound) drug concentration exceeded the MIC (%*f*T_>MIC_) and the efficacy was found through chemostat analysis. FMOX at a dose of 1 g q.i.d. was effective in preventing the growth of all ESBL-producing strains tested, even those showing high MIC against this antimicrobial (8 × MIC_90_). Based on our chemostat model, our results suggest that for FMOX, %*f*T_>MIC_ of at least 40% is sufficient to reduce >4 log_10_ CFU/mL of these bacterial populations. However, CMZ applied at its standard dose (1 g q.i.d.) against *E. coli* strains considered susceptible according to CLSI breakpoints in CLSI guideline (14) (MICs of 4–16 µg/mL) resulted in regrowth, in line with %*f*T_>MIC_ of <30%. These concentrations were insufficient to suppress bacterial growth and resulted in the emergence of resistance in only 24 h. Furthermore, CFX at 2 g q.i.d. could not suppress the growth of *E. coli* strains classified as resistant or intermediate and resulted in regrowth, with only a slight bactericidal activity against one susceptible strain of *K. pneumoniae* with <2 log_10_ CFU/mL reduction, where this antimicrobial achieved around 30% of %*f*T_>MIC_. PIPC/TAZ at 4 g/0.5 g q.i.d. showed bactericidal activity in all tested strains, including one resistant strain.

**TABLE 2:** Percentage of time the free form of flomoxef, cefmetazole, cefoxitin, and piperacillin/tazobactam above MIC and change of log_10_ CFU/mL from initial at 24 h against each strain of *Escherichia coli* and *Klebsiella pneumonia*

						Change of
			MIC	S/I/R^*a*^		log_10_ CFU/mL
Agent	Strain	ESBL^*a*^ genotype	(μg/mL)	(CLSI)^*a*^	%*f*T_>MIC^*b*^_	from initial at 24 h
Flomoxef	*K. pneumoniae* SR34688	CTX-M-15	0.12	NA^*a*^	100	−4.58
1 g	*E. coli* SR43132	CTX-M-28	0.25	NA^*a*^	100	−4.66
q.i.d.	*E. coli* SR43168	CTX-M-14	0.25	NA^*a*^	100	−4.56
	*E. coli* SR43170	CTX-M-14	0.25	NA^*a*^	100	−4.79
	*E. coli* SR53100	CTX-M-28	0.5	NA^*a*^	91.6	−4.70
	*K. pneumoniae* SR34844	CTX-M-14	0.5	NA^*a*^	91.6	−4.80
	*E. coli* SR43056	CTX-M-28	4	NA^*a*^	46.6	−4.66
	*E. coli* SR43168-CMZ exposed	CTX-M-14	4	NA^*a*^	46.6	−4.31
Cefmetazole	*K. pneumoniae* SR34688	CTX-M-15	1	S^*a*^	59.1	−4.58
1 g	*E. coli* SR43132	CTX-M-28	4	S^*a*^	28.7	3.19
q.i.d.	*E. coli* SR43170	CTX-M-14	4	S^*a*^	28.7	2.92
	*E. coli* SR53100	CTX-M-28	4	S^*a*^	28.7	2.64
	*E. coli* SR43168	CTX-M-14	8	S^*a*^	8.0	3.20
	*K. pneumoniae* SR34844	CTX-M-14	8	S^*a*^	8.0	2.27
	*E. coli* SR43056	CTX-M-28	16	S^*a*^	0	2.87
	*E. coli* SR43168-CMZ exposed	CTX-M-14	32	R^*a*^	0	2.96
Cefoxitin	*K. pneumoniae* SR34688	CTX-M-15	4	S^*a*^	32.2	−1.94
2 g	*E. coli* SR43132	CTX-M-28	16	I^*a*^	8.8	2.38
q.i.d.	*E. coli* SR43168	CTX-M-14	16	I^*a*^	8.8	1.86
	*E. coli* SR43170	CTX-M-14	16	I^*a*^	8.8	0.69
	*E. coli* SR53100	CTX-M-28	16	I^*a*^	8.8	2.32
	*E. coli* SR43056	CTX-M-28	32	R^*a*^	0	2.17
	*K. pneumoniae* SR34844	CTX-M-14	32	R^*a*^	0	2.60
	*E. coli* SR43168-CMZ exposed	CTX-M-14	64	R^*a*^	0	2.64
Piperacillin/tazobactam	*E. coli* SR43056	CTX-M-28	4/4	S^*a*^	100	−4.66
4 g/0.5 g	*E. coli* SR43132	CTX-M-28	4/4	S^*a*^	100	−4.66
q.i.d.	*E. coli* SR43168	CTX-M-14	8/4	S^*a*^	92.4	−4.56
	*K. pneumoniae* SR34688	CTX-M-15	8/4	S^*a*^	92.4	−4.58
	*E. coli* SR43170	CTX-M-14	16/4	SDD^*a*^	74.3	−4.79
	*E. coli* SR53100	CTX-M-28	16/4	SDD^*a*^	74.3	−4.70
	*K. pneumoniae* SR34844	CTX-M-14	16/4	SDD^*a*^	74.3	−4.80
	*E. coli* SR43168-CMZ exposed	CTX-M-14	>64/4	R^*a*^	<37.2	−4.31

^*a*^ S/I/R were defined based on CLSI guideline M100-Ed32 (15). ESBL, extended-spectrum β-lactamases; CLSI, Clinical and Laboratory Standards Institute; S, susceptible; I, intermediate; R, resistance; SDD, susceptible dose-dependent; NA, not available.

^*b*^ %*f*T_>MIC_, % time above MIC of free drug.

Summing up, some discrepancies between the susceptible/intermediate/resistant profiles based on CLSI breakpoints in CLSI guideline (14) and the bactericidal activity of CMZ and PIPC/TAZ were revealed through this chemostat assay. The emergence of resistant isolates seems to be associated with insufficient bactericidal activity, but FMOX showed good activity as resistant isolates did not appear. This could be the consequence of a high %*f*T_>MIC_ for FMOX, which allowed the control of resistant isolates that could have appeared during the therapy.

## DISCUSSION

It is well-known that carbapenems have excellent efficacy against ESBL-producing microorganisms, but the excessive use of these agents may result in the emergence of resistance against carbapenems. This study showed that CTX-M-9 and CTX-M-1 were the most prevalent ESBL genotypes among the clinical isolates collected in Japan during a 14 y period, which is consistent with the findings of other reports (16 - 18). The AmpC type β-lactamase genotype was observed at a very low frequency, and FMOX, CMZ, and CFX were not active against these isolates. In our chemostat model, FMOX displayed potent antimicrobial activity against these ESBL-producing isolates. Our findings regarding ESBL producers and MIC of antibiotics are consistent with several other reports on strains isolated from Japan and other countries (11, 16 - 22).

FMOX and CMZ showed a potent antimicrobial activity against most ESBL-producing *E. coli* and *K. pneumoniae* strains isolated in Japan, and the MIC_90_ values of FMOX were the second lowest, following that of MEPM. Based on the MIC distribution profile of CAZ, CFX, CMZ, and FMOX (Table 1), the percentage of susceptible isolates to CAZ was around 50%. The susceptibility percentage for CMZ (MIC ≤16 µg/mL) was around 90%, but based on this result, CMZ seemed to be effective against the isolates with MIC ≤2 µg/mL, suggesting that the susceptibility percentage could be around 50%. FMOX has been shown to be effective against isolates with MIC of up to 4 µg/mL, suggesting that the susceptibility rate was nearly 100%. This sufficient bactericidal effect of >4 log_10_ CFU/mL reduction against a resistant strain needs further investigations.

Furthermore, positive clinical outcome of MEPM and PIPC/TAZ against infections caused by ESBL-producing Enterobacterales has been reported frequently (6 - 30 - 6). The findings of the MERINO trial (31) have revealed that among patients with *E. coli* or *K. pneumoniae* bloodstream infection and CTRX resistance, definitive treatment with PIPC/TAZ compared with MEPM did not result in a noninferior 30 d mortality. Though MEPM showed good efficacy against the strains isolated in our study, carbapenem-sparing therapies are needed to reduce the risk of carbapenem resistance emergence. The combination PIPC/TAZ has been proposed in the context of carbapenem-sparing approaches, but its efficacy has been insufficient in some cases, and MEPM has been prescribed more frequently.

It has been indicated that cephamycins can be used as carbapenem-sparing therapy, but only a little preclinical and clinical information is available on their efficacy against ESBL producers (15, 32). Therefore, we evaluated the efficacy of cephamycins and oxacephem with MICs around MIC_90_ values against ESBL producers to predict their efficacy against most pathogens in clinical environments by deriving their plasma concentration curves using *in vitro* culture media. The *in vitro* pharmacodynamic trial carried out in this study recreated the variations in human blood plasma concentrations when used at the maximum clinical doses. Using multiple ESBL-producing *E. coli* and *K. pneumoniae* clinical isolates, our findings revealed that the bactericidal effects of FMOX and PIPC/TAZ were superior to those of CMZ and CFX.

We also found that it is important to ensure that appropriate dosing interval is maintained such that the drug concentration remains above its MIC (%*f*T_>MIC_) well-known pharmacokinetics/pharmacodynamics parameter for β-lactam antibiotics to be effective against the infecting pathogen (33, 34). This would ensure appropriate bactericidal effect and prevent the eventual regrowth of more resistant clones, leading to satisfactory patient outcomes.

The CLSI breakpoint for CMZ has not been updated for several years. The findings of our study reveal that the breakpoint of CMZ may not be appropriate because this compound showed no efficacy even against CMZ susceptible isolates and its %*f*T_>MIC_ did not reach 30% in most cases. The CLSI breakpoint for FMOX has not been defined yet; the breakpoint for latamoxef (4 µg/mL) is generally used as a provisional breakpoint for FMOX in Japan. Our findings reveal that FMOX needs 40% *f*T_>MIC_ to show bactericidal effect, and at this concentration, its efficacy even on bacteria against which MIC was 4 µg/mL was recorded. Considering the MIC distribution of FMOX against ESBL-producing *E. coli* and *K. pneumoniae* obtained in our study (Table 1), FMOX is expected to be effective against 97.8% (176/180) of ESBL-producing *E. coli* and 100% (33/33) of ESBL-producing *K. pneumoniae* in our collected isolates. The ratios of strains with FMOX MIC of 4 µg/mL or less among ESBL-producing bacteria in other surveys in Japan and other countries are as follows: 93.5% (29/31) of ESBL-producing *E. coli* and *K. pneumoniae* isolated from postoperative intra-abdominal infections in Japan, 92.5% (37/40) of ESBL-producing Enterobacteriaceae isolated from surgical site infections in Japan, 96.6% (112/116) of ESBL-producing *E. coli*, *K. pneumoniae*, and *Proteus mirabilis* isolated from various clinical specimens in China, and 88.6% (156/176) of ESBL-producing *E. coli* and *K. pneumoniae* from clinical isolates in Korea (12, 20, 22, 35). Therefore, FMOX 1 g q.i.d. would be effective on most ESBL-producing isolates of clinical significance obtained in Japan and other East Asian countries. The findings of our study should be further verified in *in vivo* model systems and clinical trials.

### Conclusion

There is an unmet medical need for controlling ESBL-producing bacteria effectively. Carbapenem-sparing is recommended to prevent the emergence of carbapenem resistance, and new non-carbapenem drugs are needed for controlling ESBL-producing bacteria. The results of our study suggest that maximum clinical doses of FMOX and PIPC/TAZ are expected to be effective on more than 90% of the ESBL-producing strains responsible for causing infections in Japan. FMOX showed a strong bactericidal activity against ESBL-producing bacteria, for which it is expected to become a promising treatment option, allowing the reduction in the use of carbapenems.

## MATERIALS AND METHODS

### Test strains used

During a surveillance study conducted by Shionogi & Co., Ltd., clinical isolates were collected from 15 to 17 medical centers in Japan eight times every 2 y between 2004 and 2018, and 1,274 isolates of *E. coli* and 720 isolates of *K. pneumoniae* were collected. The medical facilities here are tertiary medical institutions, and one to three facilities were selected from all eight regions in Japan. For each collection, 10 strains/facility/time for *E. coli* and six strains/facility/time for *K. pneumoniae* were collected randomly regardless of drug susceptibility and without duplication.

### Susceptibility testing and molecular characterization

The MICs of FMOX, CMZ, CFX, PIPC/TAZ, MEPM, CTRX, CFPM, and CAZ were determined using the broth microdilution method, according to the CLSI guideline M07-Ed11 (36). FMOX, CMZ, CFX, PIPC/TAZ, and MEPM, which are known to be active against ESBL-producing isolate, were selected from the viewpoint of carbapenem-sparing strategy (15). On the other hand, CTRX, CFPM, and CAZ, which are substrates of ESBL and can be degraded, were selected in order to investigate the trend of ESBL-producing pathogens in Japan and their degree of resistance to these antibiotics. CLSI breakpoints described in CLSI guideline (14) were used for the interpretation of susceptibility (susceptible/intermediate/resistant) to each isolate except for FMOX, for which CLSI breakpoints are unavailable.

Possible ESBL-producing isolates of *E. coli* and *K. pneumoniae* were selected based on the MIC of CTX or CAZ with or without clavulanic acid (in line with the CLSI guidelines) and screened for the presence of the following ESBL genes. The presence of genes encoding Ambler class A ESBL (TEM-type, CTX-M-1 group, CTX-M-2 group, CTX-M-8 group, CTX-M-9 group, and SHV-type) was assessed by polymerase chain reaction (PCR) using the commercial Cica Geneus ESBL Genotype Detection KIT2 (Kanto Chemical Co., Inc., Tokyo, Japan), followed by DNA sequencing. When the addition of 3-aminophenylboronic acid led to shifts in CAZ or CTX MIC values for ESBL-producing isolates, those strains were considered AmpC producers. The presence of genes encoding Ambler class C β-lactamases (CIT family, DHA family, FOX family, ACT family, ACC family, and MOX family) was examined by PCR using the commercial Cica Geneus AmpC Genotype Detection KIT (Kanto Chemical Co., Inc., Tokyo, Japan), followed by DNA sequencing.

### Antimicrobial activity assessed using an *in vitro* chemostat model

Five antibiotic regimes based on the maximum dosages indicated in the package insert of each commercial product (Japan) were selected (FMOX, 1 g q.i.d., 1 h infusion; CMZ, 1 g q.i.d., 1 h infusion; CFX, 2 g q.i.d., 1 h infusion; PIPC/TAZ, 4 g/0.5 g q.i.d., 0.5 h infusion; and MEPM, 1 g t.i.d., 0.5 h infusion), and the plasma variations of the corresponding active ingredients (free forms) over 24 h based on the parameters determined previously (Table S1) in Phase 1 human pharmacokinetic studies (37 - 47) and protein-binding rate of CMZ described in a package insert were recreated to compare their efficacy on five ESBL-producing *E. coli* (SR43168, SR43170, SR43132, SR53100, and SR43056) and two *K. pneumoniae* (SR34844 and SR34688) isolates. These isolates were obtained in the surveillance study described previously.

All these strains had the CTX-M-type ESBL. *E. coli* strains SR43168 and SR43170 had CTX-M-14 (CTX-M-9 group) ESBL and strains SR43132, SR53100, and SR43056 had CTX-M-28 (CTX-M-1 group) ESBL. *K. pneumoniae* strain SR34844 had CTX-M-14 ESBL and strain SR34688 had CTX-M-15 (CTX-M-1 group) ESBL.

A computer-controlled system (48) was used to recreate the expected time–concentration curves of these antibiotics (as free form) in human plasma and evaluate their effect on bacterial growth following a published protocol (49). Briefly, the test strains at 5 × 10^5^ CFU/mL were inoculated into the bacterial culture bottle containing cation-adjusted Mueller-Hinton broth (CAMHB) and continuously stirred at 37℃. The time–concentration curve of each antibiotic (as free form) was achieved by adding the antibiotics solutions and medium and collecting a portion of bacterial cultures. A control sample without any antibiotic was set with the same parameters as FMOX 1 g q.i.d., draining the bacterial culture and adding fresh medium. Sampling was performed every 2 h. The collected samples were kept at 4°C, diluted, and spread on agar plates. The number of colonies was counted the following day. The lower limit of quantitation was one log_10_ CFU/mL.

### Determination of antibiotics concentrations in the medium

Determination of FMOX, CMZ, CFX, MEPM, PIPC, and TAZ in CAMHB was performed using liquid chromatography-tandem mass spectrometry (LC/MS/MS). The supernatants obtained by protein precipitation of medium sample (20 µL) with methanol/formic acid (1,000:1, by vol., 300 µL) were analyzed by LC/MS/MS. Operating condition details were described in Table S4.

### Correlation between bactericidal effect and %*f*T_>MIC_

The percentage of time the unbound (free) form of the antibiotics tested exceeded MIC (%*f*T_>MIC_) was determined, and the correlation between this parameter and the changes in viable cell numbers after 24 h of antibiotic exposure was evaluated.

## Data Availability

The datasets generated and/or analyzed in this study are not publicly available, but are available from the corresponding author on a reasonable request.

## SUPPLEMENTAL MATERIAL

Supplemental material aac.00258-23-s0001.docx Supplemental figures and tables.

ASM does not own the copyrights to Supplemental Material that may be linked to, or accessed through, an article. The authors have granted ASM a non-exclusive, world-wide license to publish the Supplemental Material files. Please contact the corresponding author directly for reuse.
