# Supplementary material for: In vitro efficacy of humanized regimen of flomoxef against extended-spectrum β-lactamase-producing Escherichia coli and Klebsiella pneumoniae
Source: Antimicrob Agents Chemother. 2023 Aug 23;67(9):e00258-23. doi: 10.1128/aac.00258-23 (PMC10508154; doi:10.1128/aac.00258-23)

**In vitro efficacy of humanized regimen of flomoxef against extended-spectrum beta-lactamases-producing *Escherichia coli* and *Klebsiella pneumoniae***

**Supplemental Material**

**Supplemental Table S1.** Pharmacokinetic parameters used in the study

|  | V_c_ (L) | K_el_ (/hr) | K_10_ (/hr) | α | β | PB (%) |
| --- | --- | --- | --- | --- | --- | --- |
| FMOX | 7.4 |  | 2.008 | 5 | 0.78 | 35 |
| CMZ | 10.9 | 0.87 |  |  |  | 84.2 |
| CFX | 5.93 |  | 3.94 | 12.4 | 1.37 | 70 |
| PIPC | 9.7 |  | 1.15 | 3.3 | 0.801 | 16.3 |
| TAZ | 7.47 |  | 1.42 | 8.75 | 0.853 | 4 |
| MEPM | 21.5 | 0.828 |  |  |  | 2.4 |

V_c_, distribution volume; K_el_, rate constant of absorption. K_10_, elimination rate constant; α and β, macro rate constant. PB, protein binding; FMOX, flomoxef; CMZ, cefmetazole; CFX, cefoxitin; PIPC, piperacillin; TAZ, tazobactam; MEPM, meropenem.

**Supplemental Table S2.** Isolates used in the in vitro chemostat model

|  | | | |  |  |  |  |
| --- | --- | --- | --- | --- | --- | --- | --- |
|  |  |  | **MIC (μg/mL)** | | | | |
| **Species** | **Strain ID** | **Genotype** | **FMOX** | **CMZ** | **CFX** | **PIPC/TAZ** | **MEPM** |
| *Escherichia coli* | SR43168 | CTX-M-14, ST131 | 0.25 | 8 | 16 | 8/4 | ≤0.06 |
|  | SR43170 | CTX-M-14, ST131 | 0.25 | 4 | 16 | 16/4 | ≤0.06 |
|  | SR43132 | CTX-M-28, ST131 | 0.25 | 4 | 16 | 4/4 | ≤0.06 |
|  | SR53100 | CTX-M-28, ST131 | 0.5 | 4 | 16 | 16/4 | ≤0.06 |
|  | SR43056 | CTX-M-28, ST73 | 4 | 16 | 32 | 4/4 | ≤0.06 |
| *Klebsiella pneumoniae* | SR34844 | CTX-M-14 | 0.5 | 8 | 32 | 16/4 | ≤0.06 |
|  | SR34688 | CTX-M-15 | 0.12 | 1 | 4 | 8/4 | ≤0.06 |

FMOX, flomoxef; CMZ, cefmetazole; CFX, cefoxitin; PIPC/TAZ, piperacillin/tazobactam; MEPM, meropenem

**Supplemental Table S3.** Minimum inhibitory concentrations of colonies isolated from the regrowth samples of *Escherichia coli* strains exposed to cefmetazole or cefoxitin

|  | | | | | | | | | |  |
| --- | --- | --- | --- | --- | --- | --- | --- | --- | --- | --- |
|  | **MIC (μg/mL)** | | | | | | | | | |
|  | **FMOX** | **CMZ** | **CFX** | **PIPC/TAZ** | **MEPM** | **IPM** | **CAZ** | **CFPM** | **LVFX** | **AMK** |
| SR43168 (parent) | 0.25 | 4 | 16 | 32/4 | ≤0.06 | 0.25 | 16 | 16 | 16 | 2 |
| CMZ exposed | 4 | 32 | 64 | >64/4 | 0.5 | 0.12 | 8 | 4 | 16 | 1 |
| CFX exposed | 2 | 32 | 64 | >64/4 | 0.12 | 0.25 | 8 | 4 | 32 | 2 |
| SR53100 (parent) | 0.5 | 4 | 16 | 16/4 | ≤0.06 | 0.25 | 64 | 16 | 32 | 4 |
| CMZ exposed | 4 | 64 | 64 | 16/4 | 0.25 | 0.12 | 32 | 4 | 16 | 2 |
| CFX exposed | 2 | 64 | 64 | 16/4 | ≤0.06 | 0.12 | 32 | 16 | 32 | 2 |
|  |  |  |  |  |  |  |  |  |  |  |
| MIC, minimum inhibitory concentration; FMOX, flomoxef; CMZ, cefmetazole; CFX, cefoxitin; PIPC/TAZ, piperacillin/tazobactam; MEPM, meropenem; IPM, imipenem; CAZ, ceftazidime; CFPM, cefepime; LVFX, levofloxacin; AMK, amikacin | | | | | | | | | | |

**Supplemental Table S4. Operating condition of LC/MS/MS**

| HPLC conditions | | |
| --- | --- | --- |
|  | HPLC System | LC-30AD system  (SHIMADZU CORPORATION) |
|  | HPLC column | YMC Triart C18 (3 µm, 2.1 mm i.d. × 50 mm, YMC CO., LTD.) |
|  | Mobile phase A | 5mM ammonium formate /formic acid (1000:2, by vol.) |
|  | Mobile phase B | Acetonitrile |
|  | Washing solvent for rinse port | Isopropanol |
|  | Flow rate | 0.75 mL/min |
|  | Column oven temperature | 40‍°C |
|  | Autosampler temperature | 10‍°C |
|  | Injection volume | 1 µL |
|  | Run time | 1.50 min |
| Time program of mobile phase (% of Mobile phase B) | | |
|  | Initial | 5% |
|  | 1.20 min | 60% |
|  | 1.21 min | 5% |
|  | 1.50 min | Stop |
| Time program of switching valve position | | |
|  | Initial | Divert to waste |
|  | 0.40 min | Elute to mass spectrometer |
|  | 1.50 min | Divert to waste |
|  |  |  |
| Mass spectrometry conditions | | |
|  | Tandem mass spectrometer | Xevo TQ-XS (Waters Corporation) |
|  | Scan type | Multiple reaction monitoring (MRM) |
|  | Polarity | Positive, Negative (TAZ) |
|  | Ion source | Electrospray ionization |
|  | Capillary voltage (kV) | 3.2 (positive), 2.5 (negative) |
|  | Source temperature | 120‍°C |
|  | Desolvation temperature | 550‍°C |
| Monitoring ion (m/z), Cone voltage (Cone) and Collision voltage (Coll) for each analyte or IS | | |
|  | Flomoxef | Q1, 497.0; Q3, 322.8; Cone 32 V; Coll 14 eV |
|  | Cefmetazole | Q1, 471.9; Q3, 327.8; Cone 40 V; Coll 12 eV |
|  | Cefoxitin | Q1, 445.1*; Q3, 339.0; Cone 4 V; Coll 12 eV |
|  | Meropenem | Q1, 384.1; Q3, 140.9; Cone 2 V; Coll 16 eV |
|  | Piperacillin | Q1, 518.1; Q3, 142.9; Cone 10 V; Coll 20 eV |
|  | Tazobactam | Q1, 298.8**; Q3, 137.9; Cone 24 V; Coll 14 eV |

* ammonium ion addition

** negative ion

**Supplemental Figure S1.** Simulated concentration–time curves reproduced in the in vitro chemostat model and actual concentrations for each tested compound


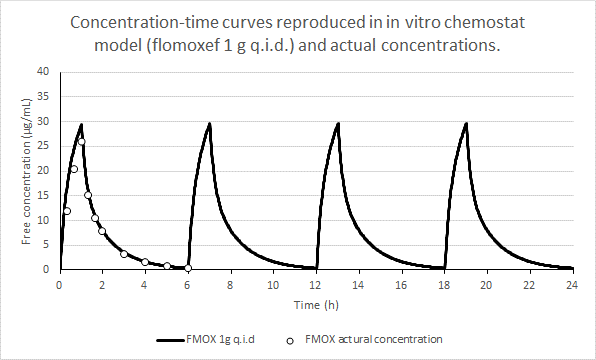


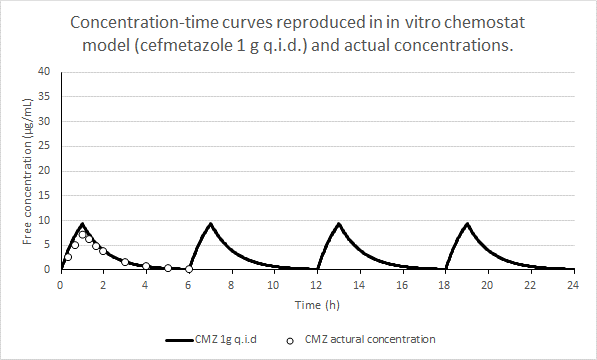


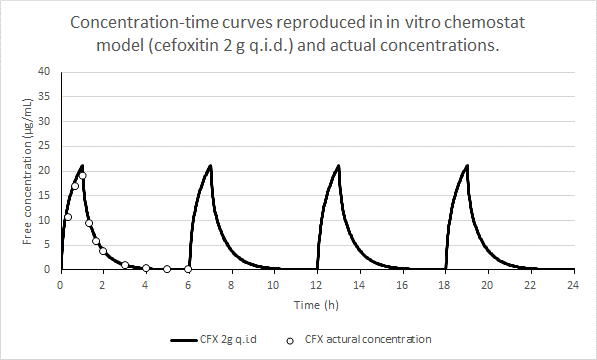


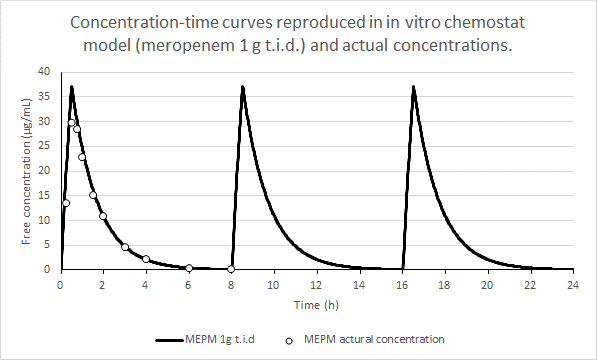


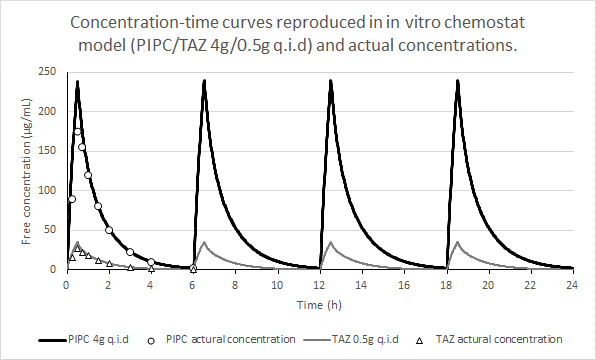

Supplement: Supplemental material — Supplemental figures and tables. [file aac.00258-23-s0001.docx]
